# Predicting toxicity through computers: a changing world

**DOI:** 10.1186/1752-153X-1-32

**Published:** 2007-12-18

**Authors:** Emilio Benfenati

**Affiliations:** 1 Istituto di Ricerche Farmacologiche "Mario Negri", Via La Masa 19, 20156 Milano, Italy

## Abstract

The computational approaches used to predict toxicity are evolving rapidly, a process hastened on by the emergence of new ways of describing chemical information. Although this trend offers many opportunities, new regulations, such as the European Community's 'Registration, Evaluation, Authorisation and Restriction of Chemicals' (REACH), demand that models be ever more robust.

In this commentary, we outline the numerous factors involved in the evolution of quantitative structure-regulatory activity relationship (QSAR) models. Such models not only require powerful tools, but must also be adapted for their intended application, such as in using suitable input values and having an output that complies with legal requirements. In addition, transparency and model reproducibility are important factors.

As more models become available, it is vital that new theoretical possibilities are embraced, and efforts are combined in order to promote new flexible, modular tools.

## Background

In this commentary, we shall discuss the recent evolution in computational methods used to predict chemical toxicity. Some of the evolutionary factors are wholly a result of scientific progress, yet others have been prompted by new demands, such as the need for regulatory models. The scientific changes to be discussed relate to the chemical information available and nature of mathematical algorithms. By looking at the European Union funded DEMETRA project, we shall see how conforming to legislative requirements can affect and influence the output and input of model. Finally, we will consider the other applications of QSAR models, and see how interactions are possible between different computational techniques, as well as *in vivo *and *in vitro *methods.

### Changes in the approaches used to predict chemical toxicity

In recent years more and more studies have been carried out in which computational programs have been used to predict the toxicity of chemical compounds. The main driving force behind this trend has been the emergence of new chemical descriptors, algorithms, and statistical perspectives, in addition to the higher expectations as to how such programs can have specific applications, such as for regulatory purposes or drug discovery. (Please note that herein, when referring to 'algorithms' we assume the classical definition, used in mathematics and information technology [1], namely: a complex calculus procedure). Another factor influencing the evolution of these approaches, although one that is perhaps less significant, is the increase in the amount of available toxicity data.

The fundamental hypothesis behind a quantitative structure-activity relationship (QSAR) model is that a chosen property (*e.g. *toxicity) can be described in relation to a chemical, which at the same time is described using certain parameters. In order to do this, we require both suitable ways of describing the chemical and a good mathematical algorithm. However, in using the same algorithm with a chemical descriptor calculated using different programs, varying results are likely. An approach close to QSAR is the so-called structure-activity relationship (SAR) model. These models express the relationship between a certain chemical property (*e.g. *fragment) and the effect (*e.g. *carcinogenicity) in a qualitative way (carcinogenic or noncarcinogenic), without assigning a continuous numerical value to the toxicity, such as a specific quantitative dose, which can have a wide range of values.

### Chemical descriptors

A few decades ago the range of chemical descriptors used was very limited. Let us take the example of Corwin Hansch's studies, in which he described the relationship between ecotoxicity and a series of parameters, including log P (where P = partition coefficient of a chemical between octanol and water) [2]. In demonstrating this relationship, it was hoped that the partition between water and the organic solvent could serve as a model to describe a fish in water. On the basis of this model, toxicity could then be understood by quantifying uptake of the compound into the fish's body. This physicochemical parameter has in fact been used in most QSAR models of aquatic toxicity [3].

Over time other descriptors have been investigated in an attempt to better explain certain factors, such as chemical reactivity and molecular size. Nowadays thousands of chemical descriptors can be calculated for a chemical structure and many fragments can be obtained using other programs [4,5].

The chemical descriptors used in older studies had a physicochemical basis, making them useful in expressing simply the biochemical mechanism responsible for the toxic effect, mainly in case of aquatic toxicity [6]. More recently different chemical descriptors and fragments have been employed, including those that are constitutional, quantum mechanical, topological, geometrical, charge related, semi-empirical, thermodynamic, and so on.

### Modelling algorithms

The growth in the number of chemical descriptors and fragments is also the result of the availability of more powerful modelling algorithms. The older QSAR models used linear equations with a very limited number of parameters, in general, one or two [5]. Multilinear regressions have now been developed, offering the possibility of screening high numbers of parameters. Non-linear models and the automatic generation of mathematical solutions have now been made possible by the emergence of other tools such as artificial neural network, fuzzy logic, and data mining algorithms [7-9]. The evolution has affected those both models that predict continuous values (regression algorithms) and those whose output is a category, for instance the toxicity class (classifiers).

However, powerful mathematical tools can produce results, which although may be formally correct, are wrong if the modeller does not evaluate the specific conditions that need to be applied, such as quality of the inputted data. Here the risk is that the model may not work when applied to new compounds, because it is only capable of replicating the toxicity of the chemicals used to construct the model [10]. This is particularly important in the case of models used for regulatory purposes, for which the OECD has developed specific guidelines (see 'Models with specific applications').

Model development involves the use of a training set that has been build up from chemical compounds whose toxicity levels are known. The model is subsequently developed using chemical parameters and a suitable algorithm. In order to ascertain whether the model is actually predictive, an assessment must be carried out. This applies to all kinds of models, from those with a single parameter, to those more complex. The possibility of chance correlation is greater when using high numbers of descriptors or parameters. Thus in order to select the most relevant descriptors, certain mathematical tools have been employed, such as principal component analysis and genetic algorithms [10].

### Statistical basis

The need for a more robust statistical basis for models has prompted discussions on how model predictivity can be assessed [11,12]. Older QSAR models were only based on the mathematical equation's fitting description, using the correlation coefficient. No proof was given that the proposed equation was useful in predicting the toxicity of chemicals not used within the study. Procedures for internal statistical validation (such as leave-one-out, y-scrambling *etc*) and external validation (employing a set of compounds not used for the model development) have been proposed. We can see that when there is limited availability of compounds, as was the case for older models, an external validation set is not possible. Thus the greater availability of toxicity data has contributed to the evolution of QSAR models; however, because of the time consuming nature of generating extra experimental data, this factor did not represented the main evolutionary driving force.

### Addressing broader targets

During the evolution of QSAR modelling more general models have been introduced that address heterogeneous chemical classes, in contrast to original QSAR approaches, which were based on classes of highly homogeneous compounds. In addition to the introduction of different technical and scientific tools, there has also been a shift in mentality regarding QSAR approaches. While the classical approach used a certain hypothesis (*e.g. *the rôle of lipophilicity) from which a model was generated, some of the new tools are based on model generation without an *a priori *hypothesis. The availability of powerful knowledge discovery techniques has allowed the exploration of a greater number of possible relationships that could not be evaluated manually, the consequence of which is that modelling activity now allows previously unforeseen hypotheses to be generated. The greater availability of toxicological data for larger sets of chemicals makes it more and more difficult to explore all possibilities manually. Of course, computers are a valuable tool in carrying out such screening.

There has been debate on the merits of classical models *versus *newer models. Those who favour the former suggest that new approaches generate models that are too complex and not sufficiently understandable. The counter argument is that what is important is having a model that is predictive. Proponents of new approaches also highlight that mechanism hypotheses have to be proved. Furthermore, even descriptors, which seem simple, are complex. For instance, log P (introduced earlier as an empirical physicochemical parameter measured from the equilibrium concentration of a chemical in a two-phase system) is actually determined using software that calculates tens of parameters, which are invisible to the final user, and whose relationship with toxicity is difficult to determine. Another example is the HOMO (highest occupied molecular orbital) energy parameter, which is calculated using complex mathematical equations, based on a series of assumptions.

Because numerous models and approaches do presently exist, it is thought that combining the results obtained from different models can surely contribute to increasing the overall reliability of the approaches used (see also 'Different perspectives on broader QSAR scenarios').

Some commercial and publicly-available models have been introduced, offering tools that can be used in diverse situations. The following are some examples:

• Many models are available on the US Environmental Protection Agency (EPA) web site that use a series of endpoints, including toxicity [13]. Properties that are predicted include log P, gas phase reaction rate, Henry's constant, melting point, boiling point, vapour pressure, biodegradation, soil absorption and water solubility. All these parameters are indirectly related to ecotoxicity. However, there are also toxicity models, such as the program ECOSAR, which predicts the toxicity of industrial chemicals to aquatic organisms (*e.g. *fish, invertebrates, algae).

• The DEMETRA project developed five, free models to determine the ecotoxicity of pesticides [14] using endpoints that include trout, daphnia, bees, and quails (oral and dietary exposure).

• There are several commercial programs with numerous endpoints, (such as carcinogenicity, mutagenicity, endocrine disruption, lethal dose for mammals, aquatic toxicity *etc*), with the following amongst those better known: TOPKAT [15], MCASE [16], HazardExpert [17], DEREK [18], TerraQSAR [19], OASIS [20].

• In the near future it is anticipated that new software will become available. The EC funded project CAESAR is developing models for five endpoints specifically related to the REACH legislation [21]. The EC funded project CHEMOMENTUM will also implement QSAR models for pesticides and industrial chemicals, taken from the DEMETRA and CAESAR projects, and presented in a more user-friendly form [22]. The so-called OECD (Q)SAR Application Toolbox will be implemented in different phases and made available to the public offering access to different databases, QSAR models and read-across tools [23,24].

### Models with specific applications

Another major change that has occurred is the development of models with a specific focus. Of course, all models address certain properties, but here we refer not to the scientific target, but the QSAR model's specific application.

Most of the published cases deal with QSAR models resulting from academic studies, for instance those studying a certain relationship between toxicity and a descriptor, proposing new descriptors as tools to better capture chemical structures, or describing a new algorithm to explore the possible links between toxicity and descriptors. Thus there are numerous models representing many possible combinations of endpoints, molecular descriptors, algorithms, and so on. Commercial software typically codifies a certain high level of modelling capability, thus providing the user with a useful tool. In this case the software has been refined and offers the prediction of several endpoints in a user-friendly environment.

When considering a model's application, we must also consider 'context'. In Europe the recent regulation concerning the Registration, Evaluation, Authorisation and Restriction of Chemicals (REACH) requires the toxicological and ecotoxicological characterisation of any chemical compound put on the market in quantities exceeding 1 ton per year [25], with further tests being requested for higher quantities. QSAR models are anticipated to play a rôle in such characterisations, limiting the need for animal experimentation, which is already restricted under the EU Directive on cosmetics [26]. Therefore in Europe there is remains much scope for debate on the possible uses of QSAR models.

Academic applications of QSAR models are the commonest. Here no strict restrictions and needs exist, beyond the interests of the scientific community. Regulatory QSAR models are more demanding because of their relationship with the law, which introduces requirements, some internal to the QSAR model process, others external. Internally the model must have a high level of quality control; whilst, externally, the model has to comply to, and be suited for, the regulatory use.

In case of predicting toxicity, QSAR models with a regulatory application should mimic the *in vivo *(and occasionally, *in vitro*) data, which are typically used in the context within legal guidelines. For instance, when a toxicity value has to be used for classification and labelling, what is important is whether the value is above or below a fixed threshold, whilst in case of risk assessment the toxicity value has to be continuous for comparison to the exposure value. Thus we can summarise the typical QSAR scheme as follows:

Data -> Information -> Knowledge

where *data *are the raw data (toxicity and chemical); *information *is the elaborated data as processed during the modelling phase; and *knowledge *is the final model output

However, when the scheme is be adapted to the 'context', it must be modified as follows:

Experimental Method -> Data -> Information -> Knowledge -> Use

where *experimental method *is the procedure used to obtain the experimental data as defined by the law; and *use *is that defined by the law for the toxicity value or class.

This modification places demands on each phase of the modelling process. For instance, the data have to refer to a certain experimental method whose uncertainty should be known, the modelling process should adhere to the thresholds identified by the law and the results should be checked against the specific application whilst also considering the number of false negatives (compounds that are predicted to be safe but are not) the uncertainty of the QSAR model, and so on. And, of course, different laws have different threshold and limits.

### Example of QSAR models with regulatory applications

In order to highlight more clearly the use of regulatory QSAR models, let us consider the European Commission-funded DEMETRA project, whose models have been developed specifically in line with the European directive on pesticides (414/91). The way in which the directive inspired the work can be summarised as follows:

• Identification of the most important endpoints: This was carried out not by the QSAR modellers, but by the stakeholders. The criteria for the selection (such as the frequency at which each endpoint is requested; number of animals used in the study; the severity/cruelty of the study; the proportion of the toxicity/exposure ration that does not trigger further testing; the availability of data *etc*) were chosen and a questionnaire was distributed to around 50 stakeholders (regulators and industry). It is worth noting here that in practically all cases a QSAR model was developed having only considered the data availability, without the other assessment evaluation carried out for the DEMETRA project.

• Identification of the regulatory guidelines: Because the models were based on the Pesticide directive, only data produced according to the protocols defined in the directive were used.

• For regulatory purposes it is fundamental to know the uncertainty of the results of the non-testing methods. Because the QSAR model is a statistical method, and thus deeply dependent on the experimental data, the uncertainty of the predicted values is necessarily related to the that of the toxicity/property data inputted to build the model. Therefore within DEMETRA the accepted uncertainty of the toxicity data was defined by regulators and stakeholders to be a factor of four. If the data uncertainty for the same chemical was higher, then the chemical was not used for the model. Here we notice that QSAR studies do not involve looking at the uncertainty of the property/toxicity value, thus highlighting the difference between typical and regulatory models.

• By comparing data held in three good databases, only reputable data were used in the DEMETRA project. The aim of this approach was not to merge chemicals, but to increase their quality using the uncertainty requirements defined earlier. Indeed, the presence of data with uncertainties higher than a factor of four resulted, for the purposes of model development, in the deletion of the chemical in question. This approach is not seen in QSAR modelling, and again shows the higher attention paid to the quality of the toxicity data employed.

• Independent researchers, using several reference sources, checked all chemical structures to be sure that they were correct; limited numbers of errors were found. Such quality control processes are typically not carried out in depth, owing to the time and other resources required.

• The full details of the algorithm were provided (in terms of toxicity data, chemical structures and descriptors, mathematical coefficients *etc*), thus making the source code available.

• DEMETRA identified the origin of the all the data (toxicity values, chemical structures, and chemical structures), as well as the access and intellectual property rights of all model components (data and models are freely available from the project's web site).

• The model's validity was confirmed by checking the model performances against a large set of compounds not used in the development of the model [27,28].

• The model's applicability domain was also defined, identifying chemical classes for which the model uncertainty was higher.

• The models specifically verified the extent and number of false negatives in order to achieve a safer regulatory application. The models were adapted to have a reduced number of false negatives. Conversely, evaluation using only squared statistical parameters (such as R^2^), which deal with false positives and negatives in the same manner, is typical in QSAR modelling. It must be noted that for regulatory purposes false negatives are very important because regulators obviously wish to avoid wrongly assigning the hazard levels of chemicals.

• DEMETRA optimised the algorithms' coefficients fixing them in the freely-available software to produce the same result in all European countries. Indeed, a model that leaves the user with freedom to modify the equation parameters is interesting from a general scientific point of view, however, is not suitable for regulatory purposes, because of its providing different results based on subjective factors.

• A detailed discussion of the application of the principles defined by the Organisation for Economic and Co-operational Development (see next section) applied to DEMETRA is available [27].

### Lessons from models with regulatory applications

As outlined, the DEMETRA strategy is one that takes into account specific requirements related to the intended regulatory application. We mentioned above that in light of legal constraints and demands, different frameworks are necessary. Further discussions on this can be found elsewhere [29,30].

A model's transparency is also a crucial factor in ensuring that a process is verifiable, something which inspired the Organisation for Economic and Co-operational Development (OECD) to define the Guidelines for the validation of regulatory QSAR models [31]. This transparency should refer to the toxicity data, the chemical structures, the chemical descriptors and the algorithms. Indeed, it may be that two different QSAR models produce different results. If certain aspects of a model are confidential (such as the toxicity values, or the algorithm employed), then the ability to determine discrepancies in the results would be reduced.

Another important feature is model reproducibility, the importance of which has already been introduced. For regulatory purposes the model uncertainty is vital – the same results should be obtained by all users (such as regulatory bodies and industries) in all countries. Indeed, there are some components in the model that affect the final predicted value. We have already mentioned the uncertainty of the inputted toxicity data, yet although this is an important factor, it is not the only one that must be taken into consideration [32]. Some models may give different results depending upon how the chemical structure has been described, or optimised, and so on. In some cases, such as for tri-dimensional descriptors, it is common to carry out a manual optimisation of the molecular coordinates, something that affects reproducibility. In general, QSAR model parameters for regulatory applications should ideally be fixed in order to maintain high reproducibility.

### Different perspectives on broader QSAR scenarios

There may be a tendency to consider regulatory QSAR models as being the principal type of QSAR model. This, however, would be inaccuarate. We earlier outlined how regulatory QSAR models have a specific application. Yet there do exist other applications that deserve attention. In other circumstances different criteria may apply, for instance although it is obvious that paying attention to the model's statistical performance is always important, the model's intended application should influence the optimisation procedure and how it is built. For instance, some models may address drug design, and in this case the model may be tailored to minimise false positives, rather than the false negatives. Indeed, for the drug industry it is important to avoid expensive experiments using chemicals that later down the line show no promise. Nevertheless, knowledge of chemical toxicity is fundamentally important in the process of drug discovery.

Models that explore biochemical mechanisms may follow different paths from those mentioned earlier, which addressed the prediction of the activity, not necessarily the mechanism. Expert modellers can explore complex situations using their experience, even if the possibility of applying the procedure further a field is limited. Industry can of course use its own confidential data for internal purposes, something that will not adversely affect the model.

The background to QSAR models is very broad. Many techniques are available, which, strictly speaking, do not refer to classical QSAR models. For instance, docking offers the possibility of studying the interactions between ligand and receptor, whilst COMFA can investigate parts of the molecule that are involved in toxicological processes [33]. While QSAR models explore the relationship between a certain chemical descriptor and a property, docking allows the introduction of specific knowledge relating to the biochemical environment in which the chemical should be active. Forces affecting chemical binding are also used in modelling. This may be useful when the property of interest is related to a biochemical process in which binding plays a key rôle. However, in cases where the process is more complex, that is, several steps are involved, appreciation of the chemical binding alone is not enough because successive (bio)chemical steps also contribute to the overall phenomenon under investigation. COMFA, for instance, is useful in identifying the steric and electrostatic factors of the molecule that affect a process.

In an evolving, complex scenario, new tools must be introduced. It is important to exhaust all possible efforts to elucidate the reasons for toxicity, as single method is sufficient to cope with such a huge task. Therefore, a wise integration of different tools is the right solution. This requires efforts to facilitate the efficient exchange of information arising from different tools, and dialogue between different components of a more complex system.

CHEMOMENTUM, mentioned earlier [22], studies GRID technology with the aim of integrating data, structures, and QSAR modelling tools, and complex models such as docking, in the same environment. The user has the option to produce models, in an automatic way, using resources located in different places. The automated workflows are based on various open-source development systems, such as OpenMolGrid. We expect that open-source tools will have a broader rôle in the near future, owing to their offering a means of increasing the range of approaches able to adapt to the evolving atmosphere we have outlined.

Another important direction for future research is how to better integrate the results obtained from different models, some of which may be conflicting. This may require a robust analysis, as demonstrated in the DEMETRA project, which developed a hybrid system for each model that integrates several sub-models [14,27].

## Conclusion

The computational methods for predicting chemical toxicity are rapidly evolving. In recent years numerous initiatives and projects have begun, and there are high expectations for the potential rôles that QSAR can play. However, further research is needed and many challenges remain in addressing the broader targets.

It is most likely that the integration of different models will become more and more important, given that it is expected that multiple models for the same endpoint will exist. The risk, of course, is that some models may yield conflicting results. The DEMETRA project has been a pioneer in this area.

It is felt that in time the use of QSAR models should become part of a broader vision, that is, by combining *in vivo *and *in vitro *methods. QSAR models are faster and less expensive and ought to be used as the first step on this process. Feed-back procedures should also be planned in order to better integrate tests with non-testing methods. The EC funded project, OSIRIS, is working on integrating different methods in line with the REACH legislation [34], further discussion of which can be found on the RAINBOW project website [35].
